# ­­Presence and distribution of 14-3-3 proteins in human ocular surface tissues

**Published:** 2008-12-31

**Authors:** Jwalitha Shankardas, Michelle Senchyna, Slobodan D. Dimitrijevich

**Affiliations:** 1 Graduate School of Biomedical Sciences, UNT Health Science Center, Fort Worth, TX; 2 Alcon Laboratories, Inc., Fort Worth, TX; 3 Department of Integrative Physiology, UNT Health Science Center, Fort Worth, TX

## Abstract

**Purpose:**

14–3-3 is a highly conserved, ubiquitously expressed family of proteins. At least seven mammalian isoforms (β, ε, γ, η, θ, σ, and ζ) are known. These proteins associate with over 200 different target molecules and activate several downstream signaling cascades involved in the regulation of metabolism, cell cycle, apoptosis, protein trafficking, transcription, stress responses, and malignant transformations. We are interested in the role of these proteins in the mechanisms regulating homeostasis and the pathologies of the human ocular surface. Therefore, our purpose is to determine the expression of the 14–3-3 proteins in the human cornea, the conjunctiva, and the primary cells comprising these tissues.

**Methods:**

Using immunofluorescence, we determined the expression of 14–3-3 β, ε, γ, η, θ, σ, and ζ in paraffin sections of the human cornea and conjunctiva. Using indirect immunofluorescence and western blot analysis, we also determined the expression of these isoforms in primary corneal epithelial cells, keratocytes, endothelial cells, and primary conjunctival epithelial cells. The expressions of these isoforms in primary epithelial and endothelial cells were compared with the same expressions in several corneal cell lines. Western blot analysis was used to determine the presence of 14–3-3 isoforms in the culture medium from corneal epithelial cells, cell lines, and the tear fluid.

**Results:**

All the 14–3-3 isoforms were expressed in the corneal and conjunctival epithelia as well as primary epithelial cells and cell lines. Expression of 14–3-3 σ was confined to epithelial cells and was secreted into the culture medium of primary cells and cell lines. We also report for the first time that two of the secreted isoforms, 14–3-3 γ and ζ, are also present in the human tear fluid.

**Conclusions:**

We have determined that all the mammalian 14–3-3 isoforms are expressed in the human cornea, conjunctiva, and the component cells and that the 14–3-3 σ isoform was found to be epithelial cell specific. We propose that the intracellular and extracellular presence of 14–3-3 σ suggest its involvement in the epithelia specific signaling pathways.

## Introduction

The 14–3-3 (FTT, which stands for fourteen-three-three) proteins, discovered in the central nervous system (CNS) and cerebrospinal fluid (CSF) in 1967, make up a family of highly conserved acidic molecules [1]. Seven mammalian isoforms (β, γ, ε, σ, ζ, θ, and η) are known, and each is the product of a separate gene. Although phosphorylation is not considered to be an essential requirement for their biological activity, FTT α, δ, and τ are the phosphorylated β, γ, and θ isoforms [2-4]. The number of eukaryotic cells and tissues in which these proteins have been detected continues to increase, suggesting a ubiquitous expression and function [4]. The biological activity of this family of proteins is associated primarily with homodimers, but the heterodimers are beginning to be observed and studied [5]. The FTT dimers interact with over 200 known target molecules phosphorylated at specific serine or threonine residues, but the interactions that do not involve phosphorylated partners have also been identified [6-10]. The interactions of FTT proteins with their partners are components of the following general mechanisms: i) conformational change in the binding partner, ii) masking or exposure of the functional motifs that regulate the intracellular localization of partner molecules, and iii) changes in the phosphorylation state or stability of the target molecules [3]. The diverse consequences of these interactions include events associated with cell cycle control [4,11], metabolism [4], apoptosis [5], protein trafficking [4], transcription [4], stress responses [12], and malignant transformation [4,5,11,12]. However, this is still a relatively new and rapidly developing field of study, and many regulatory signaling pathways involving FTT proteins remain poorly understood or unknown. Most of the biological activities of FTT proteins that have so far been characterized are concerned almost exclusively with intracellular events. The discovery that FTT σ isoform is secreted suggests involvement in autocrine or paracrine events and creates interesting possibilities for extracellular FTT functions [13-18].

Considering the scope of the involvement of FTT proteins in the functions of living systems, it is not surprising that studies cover diverse areas of interest ranging from embryonic development to cancer biology. Of particular interest in cancer biology is the importance of FTT σ in a variety of cancers (breast cancer [19], carcinomas of the urinary bladder [20], ovaries [21], prostrate [21], and salivary glands [22]), suggesting that this isoform might be an oncogene [23]. Studies of the role of FTT proteins in embryonic development have been facilitated by the knock out/knock down animal models (murine) [24-26] or mutations in FTT genes [25]. The mutation in FTT *σ*, which gives rise to the repeated epilation (Er) mouse, is lethal in homozygous animals. The heterozygous mice (Er/+) survive and are a very useful research tool for the characterization of DNA alterations and the studies of global and tissue specific consequences [25].

We are particularly interested in the events that are involved in the homeostasis, repair, and pathologies of the cornea and conjunctiva. The epithelia in these dynamic barrier tissues are particularly tightly regulated to achieve the balance of cellular events that maintain proper tissue functions. The cell cycle regulation in these tissues may therefore be considered a critical factor that maintains the appropriate equilibrium between tissue specific proliferation and differentiation. A significant component of these equilibriums is the presence of the putative stem cell compartment located in the limbus [27,28]. Numerous intracellular and extracellular effectors are involved in epithelial cell cycle regulation, but the role of FTT proteins in this context has not been studied. We here describe the first systematic FTT profiling of the human cornea, conjunctiva, primary cells, and some cell lines by determining the expression of all seven mammalian isoforms using indirect immunofluorescence and western blot analysis. We also show that FTT σ expression is specific for corneal and conjunctival epithelia and that FTT σ, γ, and ζ, are secreted into the culture media of the primary corneal and conjunctival epithelial cells and some corneal epithelial cell lines. Furthermore, the secreted γ and ζ isoforms were found in the human tear fluid from normal volunteers, but FTT σ was not found.

## Methods

All samples were obtained in compliance with good clinical practice with informed consent from the participants under institutional review board regulations and in accordance with the tenets of the Declaration of Helsinki.

### Cell culture

#### Primary corneal and conjunctiva epithelial cells

Epithelial sheets were obtained from Eye Bank (Lions Eye Institute for Transplant & Research, Inc., Tampa, FL) corneas (after removal of residual sclera and conjunctiva tissue) and conjunctival tissue (primarily bulbar), and the primary cells were cultured as previously described [29-31]. Briefly, donor tissue was incubated in dispase (BD Biosciences, San Jose, CA), diluted to 12 units/ml with calcium free EpiLife® medium with human corneal growth supplement (Cascade Biologicals, Portland, Oregon) at 4 °C for 48 h. The epithelial sheets were removed from the stromas, dissociated into single cell suspension, and then plated into tissue culture (TC) flasks (75 cm^2^, vented). Flasks were coated with murine collagen type IV (at a concentration of 5 μg/ml) using a cell scraper to evenly coat the entire growth surface. The coated flasks were air-dried under sterile conditions before use (or stored sterile at 4 °C). The cells were cultured in serum free defined medium (EpiLife®, Cascade, Portland, OR) until approximately 80% confluent.  The cells were then subcultured in EpiLife® by harvesting with trypsin EDTA (Gibco, BRL, Carlsbad CA), neutralization of proteolytic activity with trypsin inhibitor (Sigma-Aldrich, St. Louis, MO), and plating into TC flasks freshly coated with collagen type IV (BD Biosciences, San Jose, CA).

#### Human corneal epithelial cell lines

Corneal epithelial cell lines, SV40 [32] (a kind gift from Alcon Laboratories, Inc.) and E6/E7 immortalized [33] (a kind gift from Dr. Vinod S. Mootha; Department of Ophthalmology, University of Texas Southwestern Medical School, Dallas, TX), were thawed from frozen stock and cultured in serum free defined media (EpiLife®, Cascade Biologicals) to 80% confluence. The epithelial cells were subcultured by harvesting with trypsin/EDTA (Gibco BRL), neutralizing the proteolytic activity with trypsin inhibitor (Sigma-Aldrich), and plating into freshly collagen IV coated TC flasks (BD Biosciences).

#### Corneal stroma fibroblasts

The epithelium was removed from the donor corneas as described above, and the residual endothelium was peeled off with Descemet’s membrane. The denuded stromas were minced into 2 mm cubes, which were allowed to adhere to TC flasks (or six well TC plates). To these pieces, medium DMEM (Gibco BRL) containing 10% fetal bovine serum (FBS; Atlanta Biologicals, Lawrenceville, GA) was then carefully added. When the outgrowth of the corneal fibroblasts (keratocytes) was evident (within a week from set up), the pieces were removed and the culture process continued. The cells were subcultured into fresh TC flasks as needed.

#### Corneal endothelial cells

Primary endothelial cells were cultured as previously described [34]. Briefly, the Descemet’s membrane bearing endothelial cells was removed from donor corneas and the whole endothelial explants cultured in DMEM containing 10% FBS. The E6/E7 transformed endothelial cells were thawed from frozen stock and cultured in DMEM supplemented with 10% FBS.

### Immunocytochemistry

Approximately 15,000 cells were plated on glass coverslips (12 cm^2^, Thermofisher; Fisher Scientific, Pittsburgh, PA) and cultured in their respective media. When the cultures had stabilized, the coverslips were rinsed in PBS and fixed/permeabilized in methanol:acetone (1:1) for  10 min at −20 °C. After rehydration in phosphate buffered saline (PBS; 0.256 g/l NaH_2_PO_4_ H_2_O, 1.19 g/l Na_2_HPO_4_, 8.76 g/l NaCl, pH 7.4) for 30 min and three distilled water washes, the cells were blocked overnight at 4 °C in PBS+1% bovine serum albumin (BSA). The cells were then rinsed with PBS and distilled water three times (3X) and incubated at 4 °C overnight with primary (1°) antibody diluted in PBS. After rinsing three times 10 min each (3X 10 min) in PBS containing Tween-20 (0.1%), cells were incubated with secondary (2°) antibody at room temperature (RT)  for 1.5 h and rinsed in PBS+Tween-20 (0.1%, 3X 10 min). The specimens were rinsed in PBS (3X 10 min), distilled water for 30 min, stained with 4’,6-diamino-2-phenylindole (DAPI), and mounted on glass slides (FluorSave™; Calbiochem, La Jolla, CA).

### Immunohistochemistry

Donor corneas and conjunctiva were fixed in 4% formaldehyde at 4 °C for 24 h, dehydrated through a series of ethanol and xylene incubations, and embedded in paraffin. Embedded tissues were sectioned (~10μ thickness), and sections were deparaffinized by incubations in xylene and ethanol. After rehydration for 30 min in PBS, distilled water washes (3X), and blocking overnight at 4 °C in PBS+1% BSA+1% horse serum, specimens were rinsed with PBS and distilled water (3X) and incubated with 1° antibody at 4 °C overnight. After rinsing in PBS (3X 10 min) containing 0.1% Tween-20, the tissue sections were incubated with 2° antibody at RT for 1 h and rinsed in PBS+0.1% Tween-20 (3X 10 min). The specimens were rinsed in PBS (3X 10 min) and distilled water (1X 10 min), and glass slides were mounted on the sections after DAPI staining (FluorSave™, Calbiochem).

### Antibodies

Commercially available anti-FTT primary antibodies were purchased from Santa Cruz Biotechnology (Santa Cruz, CA), which were directed to either the NH_2_– or COOH-terminus or an internal epitope, and were used in the dilution recommended by the supplier. One anti-FTT σ antibody was from LabVision (Freemont, CA) and was directed against an unknown epitope. Alexa Fluor 594 nm goat anti-mouse, Alexa Fluor 594 nm goat-anti-rabbit, and Alexa Fluor 594 nm donkey anti-goat (Molecular Probes/Invitrogen, San Diego, CA) secondary antibodies were used at dilutions of 1:1,000. Negative controls in all experiments were specimens labeled with 2° antibody only and DAPI to show the nuclei; these showed virtually no background fluorescence.

### Image acquisition

Mounted specimens were examined on Olympus AX70 fluorescent microscope (Olympus, Center Valley, PA) using SPOT^®^ TWAIN software (Microsoft, Issaquah, WA).

### Western blot analysis

Cultured cells, whole epithelia sheets, and endothelial cells were treated with lysis buffer (2.5 ml 1 M Tris buffer [pH=7.0], 1 g SDS, and 2.5 g sucrose in 50 ml distilled water) for 5 min at RT. Genomic DNA was sheared by several passes through a 22 gauge needle, and samples were stored at –20 °C until needed. BCA (bicinchoninic acid) protein assays (Pierce, Rockford, IL) of lysates were performed to determine the protein concentration and to ensure equal loading of lanes. SDS-PAGE was performed at RT, and 20 μg protein/lane were loaded with 12% Tris-glycine at 150 V with Tris/glycine as the running buffer. Protein bands were transferred onto nitrocellulose membranes (VWR International, Irving, TX) by electroblotting overnight (4 °C) at 10 V in Tris/glycine buffer with 20% methanol and confirming the transfer with Ponceau Red staining (Sigma-Aldrich) of the membranes. After de-staining in distilled water, membranes were incubated in blocking buffer (5% powdered milk and 1% BSA in PBS) for 1 h at RT. Membranes were then incubated with 1° antibody for 30 min at RT and then overnight at 4 °C, and they were incubated again the following morning for 30 min at RT. After rinsing in PBS containing 0.1% Tween-20 (3X 10 min), the membranes were incubated with 2° antibody for 1 h at RT, rinsed in PBS with 0.1% Tween-20 (3X 10 min), and developed (ECL Chemiluminescence; Amersham Biosciences, Little Chalfont, England).

### Tear fluid collection

Tear washes were collected from three normal subjects as previously described [35,36]. The samples were obtained in compliance with good clinical practice, with informed consent under institutional review board regulations, and in accordance with the tenets of the Declaration of Helsinki. Sterile saline was instilled onto the donor ocular surface. Subjects were then asked to move their eyes without blinking, and tears were collected using a micropipette. Cellular debris was then removed by centrifugation. Approximately 20 μg of protein was then loaded directly onto the SDS-PAGE gel for electrophoresis and subsequent western blot analysis.

### Detection of secreted 14-3-3 isoforms

Conditioned medium from primary corneal and conjunctival epithelia cells, as well as E6/E7 and SV40 transformed corneal epithelial cells was collected after 48 h of culture. The conditioned medium was centrifuged to remove any cellular debris. Strataclean® resin (Stratagene, La Jola, CA) was used to pull down protein from the conditioned medium as per the manufacturer’s instructions. Briefly, after the addition of 1 μl/ml resin, the conditioned medium was centrifuged at 10,000 rpm to pellet the resin. The resin was then re-suspended in 10 μl of sample loading buffer. The samples were then loaded directly onto the SDS-PAGE gel for electrophoresis and subsequent western blot analysis.

## Results

### Expression of FTT isoforms in the tissue sections of human cornea and conjunctiva

**Figure 1:**
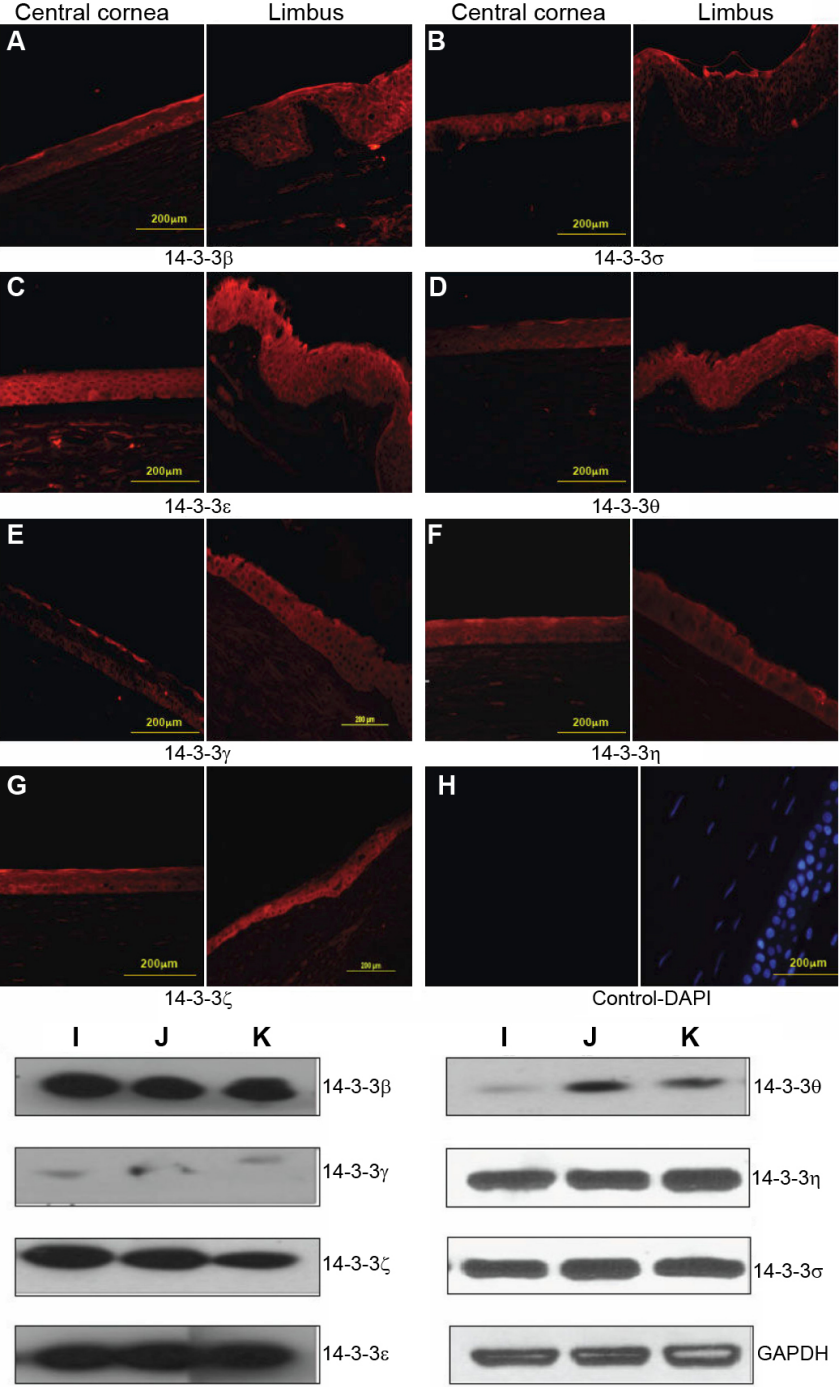
Expression of 14-3-3 proteins in the human cornea. Panels **A**-**H** show that all the 14-3-3 isoforms are expressed in the cornea while the 14-3-3σ isoform is restricted to the epithelium. Comparison of expression of 14-3-3 isoforms in the primary corneal cells with that in cell lines as determined by western blot analyses of whole cell lysates. Strong expression of 14-3-3 β, η, ε and σ is shown in the primary corneal epithelial cells (**I**), E6/E7 transformed corneal epithelia cells (**J**), and SV40 transformed corneal epithelia cells (**K**).  The expression of 14-3-3 θ is barely detectable in primary corneal epithelial cells (**I**) and but is present in the E6/E7 and SV40 transformed cells (**J** and **K**). The expression of 14-3-3 γ is very weak in the three cell types (**I**, **J**, and **K**).

**Figure 2:**
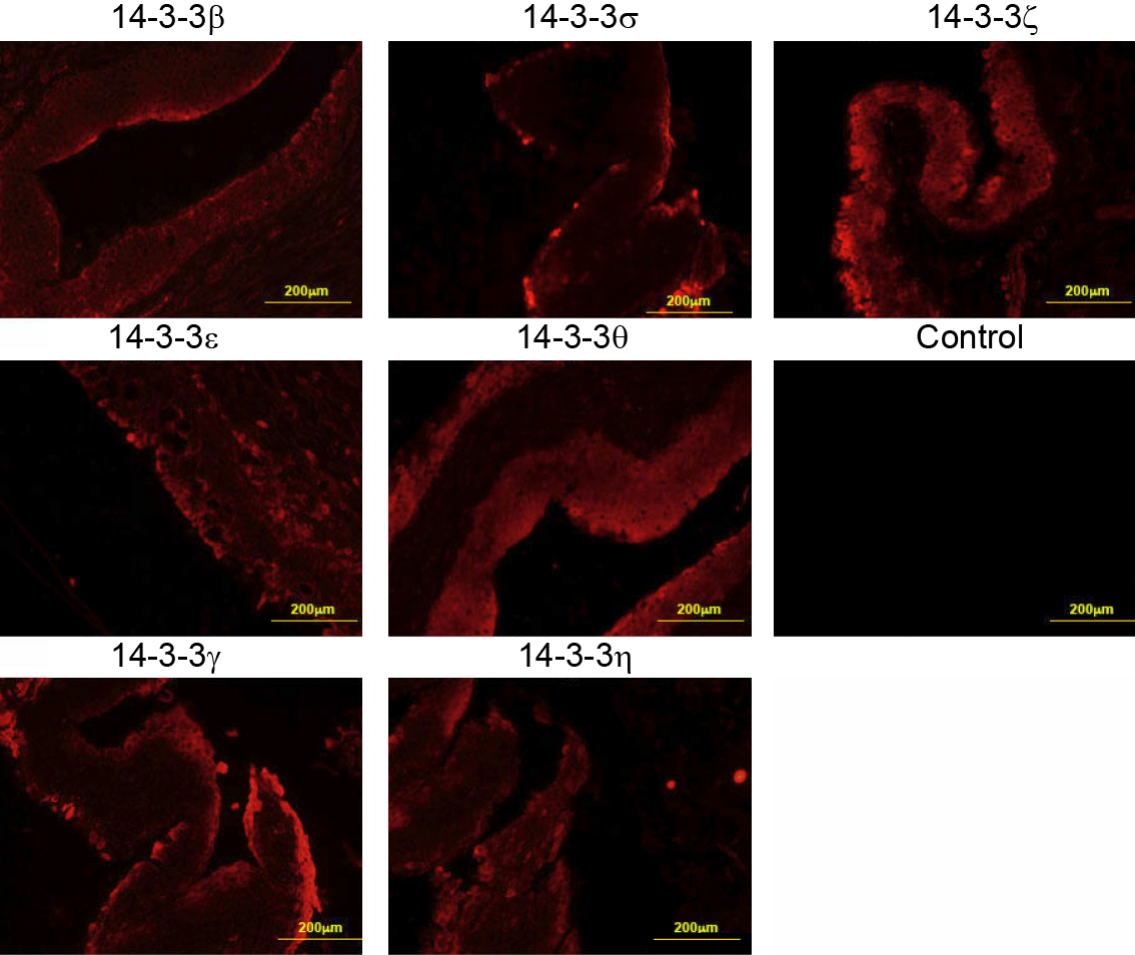
Expression of 14-3-3 proteins in the cross-sections of the conjunctival whole tissue as determined by indirect immunofluoresence. The panels show that all the 14-3-3 isoforms are expressed in the cornea while the 14-3-3 σ isoform is restricted to the epithelium.

Using commercially available antibodies directed against FTT β, ε, γ, η, θ, σ, and ζ, the tissue sections of paraffin embedded human corneas and conjunctivas (3 sections from 3 donors for each tissue) and the qualitative expression of all FTT isoforms was determined. Six FTT isoforms (θ, γ, η, β, ζ, and ε) are expressed throughout the central cornea and the limbus (Figure 1A,C,D-G). However, the expression of FTT σ is confined to the epithelium (Figure 1B). Specifically, FTT σ appears strongly expressed in the superficial layers of the central corneal epithelium and the superficial/suprabasal limbus epithelium. FTT β, γ, and η appear strongly expressed in the superficial layers in the central corneal epithelium and uniformly expressed throughout the limbus epithelium (Figure 1A,E,F). FTT ε and ζ are uniformly expressed throughout the central cornea and the limbus epithelia (Figure 1C,G). FTT θ is uniformly expressed in all the epithelial cell layers (Figure 1D). All seven FTT isoforms are expressed in the human conjunctiva, and FTT σ is only expressed in the conjunctival epithelium (Figure 2). There was also no significant donor-to-donor variability (n=3) in the samples that were used in the study.

### Expression of FTT isoforms in the primary cells of the cornea and corneal cell lines and primary conjunctival epithelial cells

**Figure 3:**
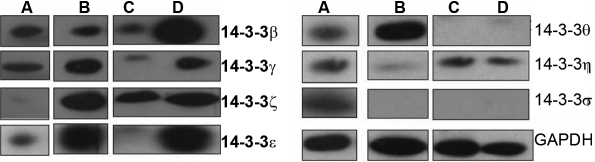
Comparison of the expression of 14-3-3 isoforms in the primary corneal and conjunctival cells with that in cell lines as determined by western blot analyses of whole cell lysates. All the isoforms are expressed in the primary conjunctial epithelial cells but the 14-3-3 ζ is expressed weakly (**A**). Primary corneal stromal fibroblasts express all the 14-3-3 isoforms but the expression of η isoform is low, and σ isoform is not expressed (**B**). The primary corneal endothelial cells express all 14-3-3 isoforms except σ and θ is barely detectable (**C**). The E6/E7 transformed corneal endothelial cells express all the 14-3-3 isoforms more robustly than the primary cells, the expression of θ isoform is more evident and there is no expression of σ isoform (**D**).

**Figure 4:**
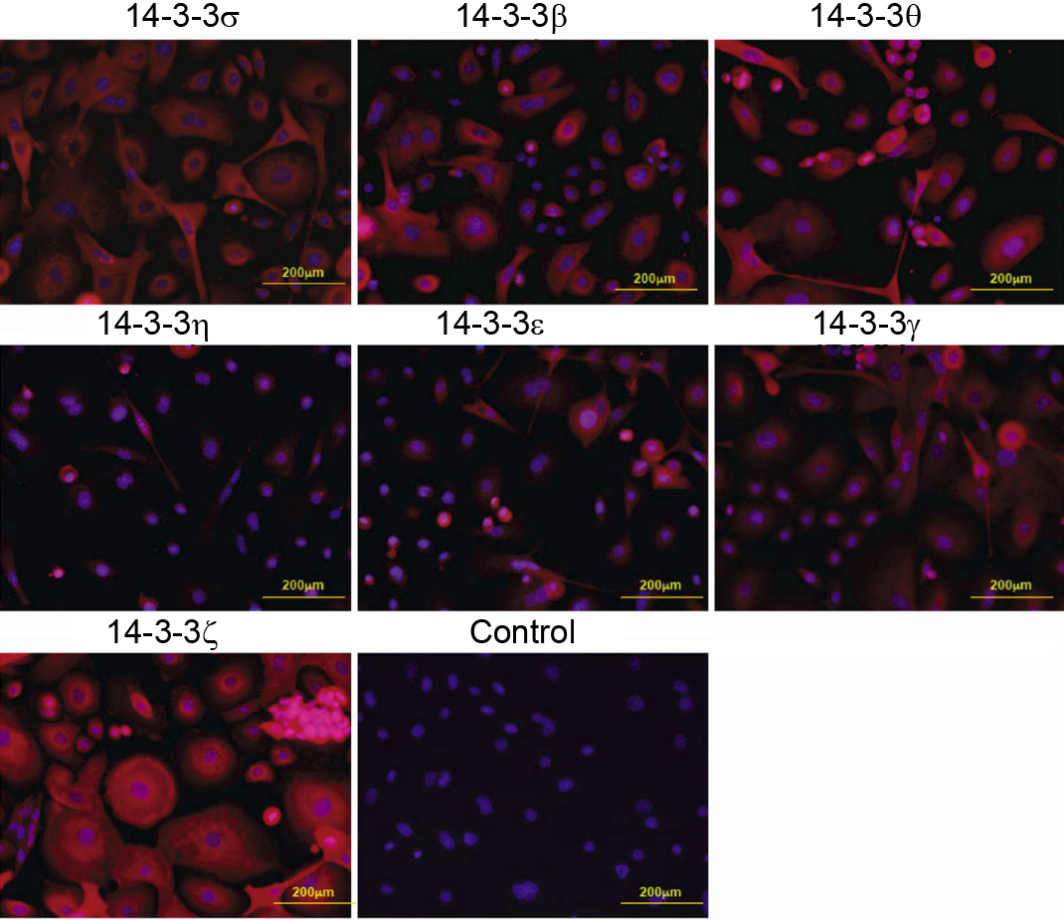
The expression of 14-3-3 proteins in the cultured primary corneal epithelial cells as determined by indirect immunofluorescence. The panels show that all the 14-3-3 isoforms are expressed in the cultured primary corneal epithelial cells.

**Figure 5:**
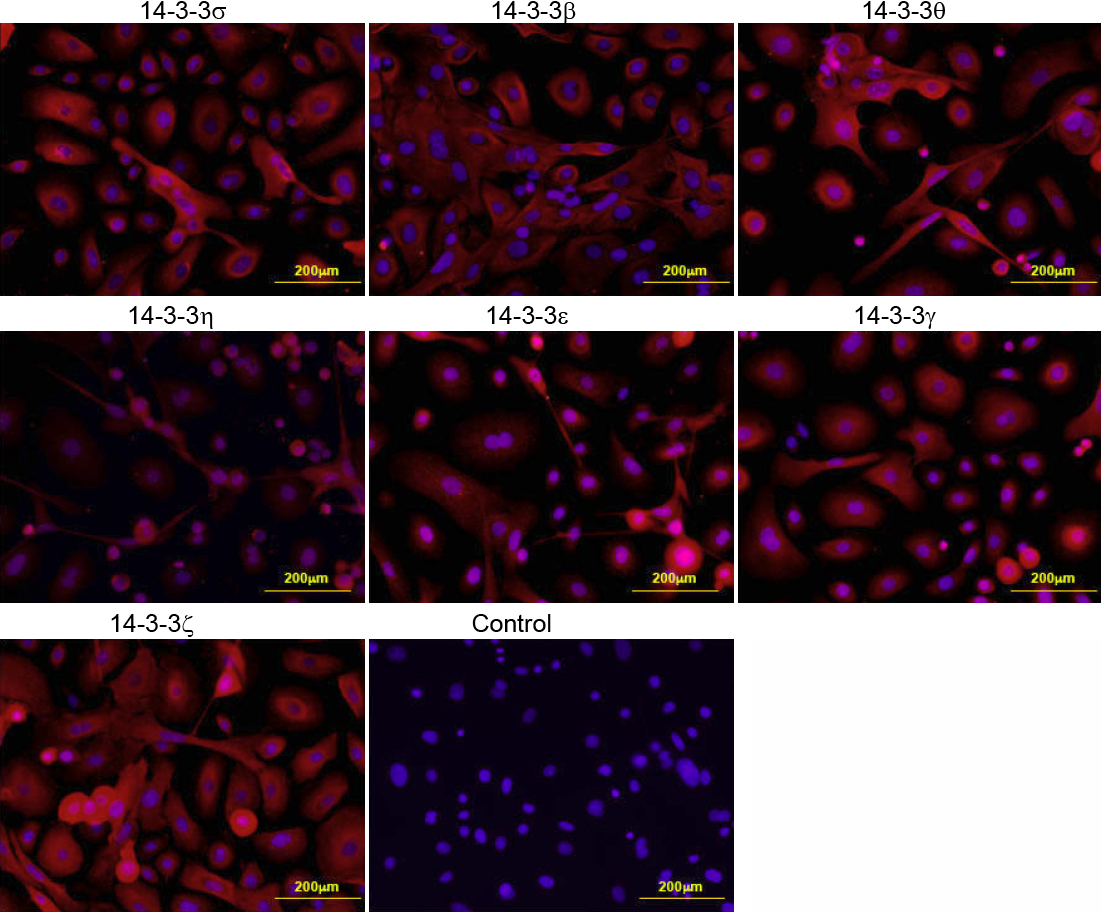
The expression of 14-3-3 proteins in the cultured primary conjunctival epithelial cells as determined by indirect immunofluorescence. The panels show that all the 14-3-3 isoforms are expressed in the cultured primary conjunctival epithelial cells.

We then examined the expression of FTT proteins in cultured primary cells of the cornea and conjunctiva and two human corneal epithelial cell lines. The results of western blot analyses (shown in Figure 1I-K and Figure 3) were quantified and summarized in Table 1. The results of the immunocytochemical analysis are shown in Figure 4 and Figure 5. All of the FTT isoforms are expressed in the primary corneal and conjunctival epithelial cells. The corneal epithelial cells immortalized by E6/E7 and SV40 transfection expressed all seven FTT isoforms at varying levels (Figure 1I-K and Table 1). FTT ζ expression is high in the primary corneal epithelial cells and the cell lines, but the expression of FTT γ is very low. FTT θ expression is higher in both transformed corneal epithelial cell lines than in the primary cells. Primary corneal stroma fibroblasts (keratocytes) express all FTT isoforms except FTT σ, and FTT β is weakly expressed while FTT η is barely detectable (Figure 3, Table 1). Primary corneal endothelial cells express all FTT isoforms except FTT σ. FTT θ expression is low (Figure 3, Table 1) in primary and E6/E7 transformed corneal endothelial cells. The latter show a higher expression of FTT ε, γ, and β than the primary cells. The conjunctival epithelial cells express all seven FTT isoforms, but FTT ζ expression is barely detectable (Figure 3, Table 1).

**Table 1:** Expression of 14–3-3 isoforms in the cells of the ocular surface.

**FTT isoforms**	**Corneal epithelial cells**			**Conjunctival epithelial cells (Wt)**	**Corneal stroma fibroblasts (Wt)**	**Corneal endothelial cells**	
	**Wt**	**E6/E7**	**SV- 40**			**Wt**	**E6/E7**
σ	++	++	++	++	-	-	-
β	++	++	++	++	+	+	++
γ	+	+	+	++	+	+	++
θ	+	++	++	+	+	+	+
ζ	++	++	++	+	+	+	+
η	+	+	+	+	+	+	+
ε	++	++	++	+	+	+	++

Expression levels as determined by western blot analysis. Densitometric value (after normalization) of 0.5 or greater is symbolized by “++” (very high expression). A value less than 0.5 is denoted as “+” (moderate expression), and value of 0 is represented as “-” (little or no expression). Antibodies were purchased from Santa Cruz Biotechnology: 14–3-3 σ (sc-7381, antibody directed against an epitope in the NH_2_-terminus and sc-7683 directed against an epitope in the COOH-terminus were both used  individually); 14–3-3 β (sc-629, antibody directed against epitope in the NH_2_-terminus); 14–3-3 γ (sc-731 antibody directed against epitope in the COOH-terminus); 14–3-3 θ (sc-732 antibody directed against epitope in the COOH-terminus), 14–3-3 ζ (sc-1019 antibody directed against epitope in the internal sequences), 14–3-3 η (sc-17286 antibody directed against epitope in the internal sequences), and 14–3-3 ε (sc-1021 antibody directed against epitope in the internal sequences).

### FTT isoforms secreted by cultured corneal and conjunctival epithelial cells

**Figure 6:**
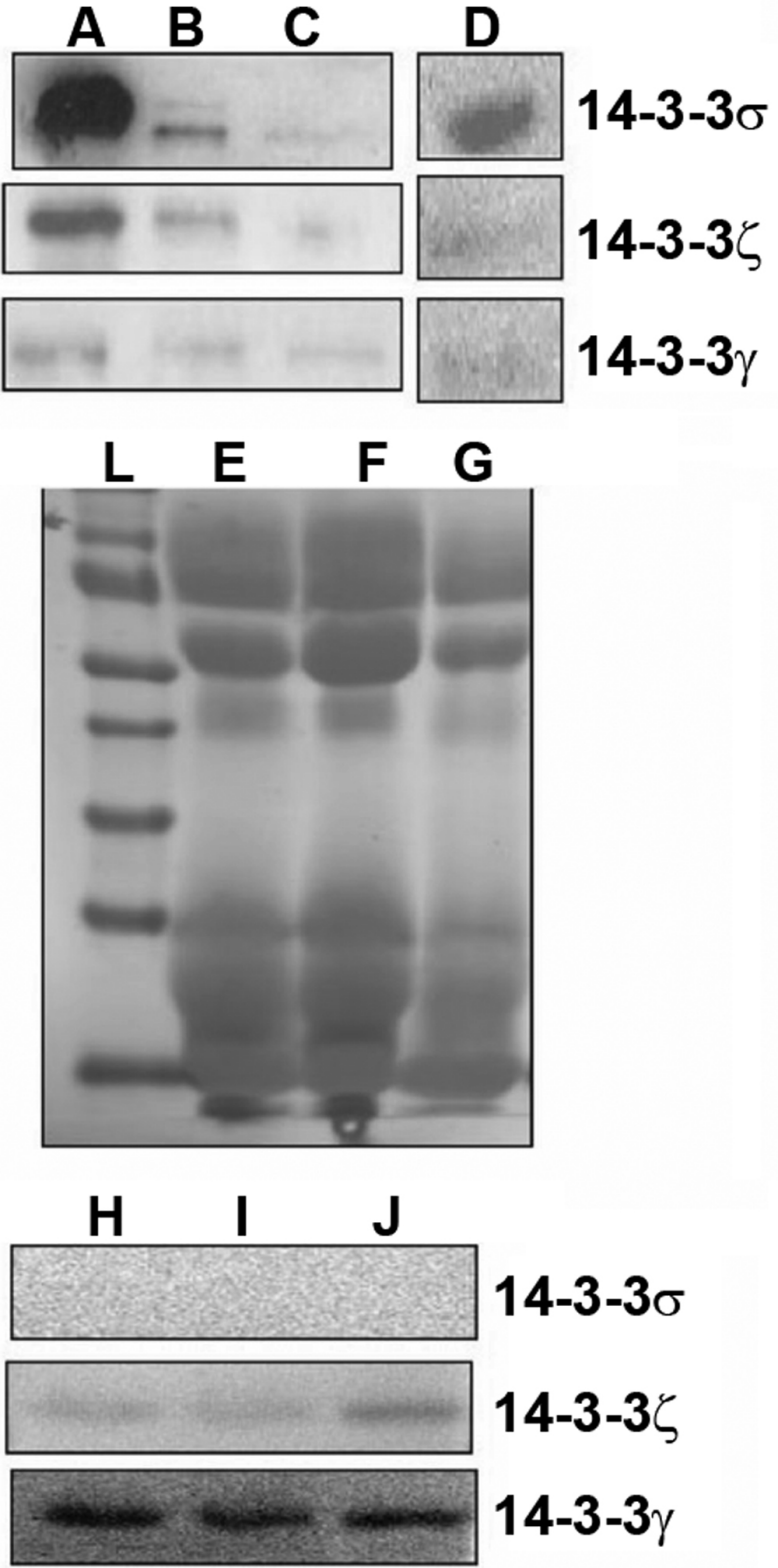
Western blot analysis of the conditioned medium from the corneal epithelia cells and cell lines and human tear fluid. The primary corneal epithelial cells were shown to secrete 14-3-3 σ, ζ, and γ in to the condition medium (**A**). E6/E7 and SV 40 transformed corneal epithelial cells and primary conjunctival epithelial cells also secrete these three isoforms (**B**, **C**, and **D**, respectively). The human tear fluid from normal human volunteers shows the presence of 14-3-3 η and γ isoforms but sigma is absent (**H**, **I**, and **J**). **E**, **F**, and **G** are derived form a Ponceau Red stained blot to show protein loading. The molecular weight ladder is seen in **L**.

Corneal and conjunctival epithelial cells when cultured in the serum free defined medium (EpiLife) were shown to secrete FTT σ, γ, and ζ into the culture medium (Figure 6A). Western blot analysis was used to show that transformed corneal epithelial cells also secrete these three FTT isoforms. EpiLife culture medium was used as a negative control.

### Presence of FTT isoforms in the human tear fluid

We then examined tear fluid from normal volunteers for the presence of the FTT isoforms that were shown to be secreted into the culture medium of corneal and conjunctival epithelial cells. Western blot analysis of tear samples from three donors showed that while FTT γ and ζ isoforms were present, the FTT σ was absent from the tear fluid (Figure 6C).

## Discussion

We have been interested for some time in the human ocular surface tissue [37-40]. The diversity of cellular events, specifically the balance between the cell cycle regulation and differentiation/stratification of the self-renewing epithelia, is a feature in their homeostasis and repair. We therefore consider that the diverse biological activities of the FTT family of proteins may play a significant role in the human cornea and conjunctiva. While studying protein expression in corneal dystrophies, the presence of 14–3-3 σ in the corneal epithelium has been reported [18]. For the first time, our results show that all the FTT isoforms are expressed in the cornea and conjunctiva but that the expression of FTT σ, also known as stratifin (*Sfn*), is confined to the differentiated superficial layers of the corneal epithelium. This correlates well with the expression of stratifin in the epidermis, a stratified epithelium of the skin [41-43]. Although this FTT isoform is proposed to be associated with differentiation, its presence and distribution in the stratified epithelia other than skin has not been studied and its role in differentiation has not been established.

The abrogation of FTT σ in primary keratinocytes has lead to immortalization [39,40], and its expression is reduced in some cancers [20,21,44] but upregulated in others [45-47]. To begin a detailed study of this proposed relationship with cell cycle control, we compared the expression profile of all the isoforms in the primary corneal epithelial cells with that in two immortalized cell lines in which the cell cycle arrest has been altered by E6/E7 and SV40 transduction (see Table 1). In this context, SV40 and E6/E7 immortalized cells showed levels of expression of all seven isoforms (including FTT σ) similar to those determined for primary cells, with the exception of the low levels of FTT γ expression, from western blot analysis. The isoform that was “differentially” expressed was FTT θ. It was barely detectable in the primary cells but expressed robustly by both the corneal epithelial cell lines. This FTT isoform has been proposed to be involved in nuclear localization of telomerase [48], which is inactive in all somatic cells, particularly as they enter senescence. Endogenous expression and activity of human telomerase reverse transcriptase (hTERT) has been detected in most cancer cells, and its ectopic activity has been shown to extend the proliferative in vitro life span when constitutively expressed in several types of somatic human cells [49-53]. We show a low expression of FTT θ in the primary corneal endothelial cells, which are not particularly mitotic in spite of having long telomeres [54]. The antibody to FTT θ [55] and the DNA fragments of the FTT σ promoter region [56] have been detected in the blood of newly diagnosed lung cancer patients and breast cancer patients, respectively, suggesting a link with cell cycle dysregulation. The role of FTT σ in corneal epithelial homeostasis appears to be more complex than its role in the differentiation of keratinocytes and the homeostasis and pathologies of the epidermis.

We also determined the expression of FTT proteins in conjunctival tissue. Consistent with the expression profile shown in the cornea, all the FTT isoforms were expressed in the epithelium and stroma of the conjunctiva, and FTT σ expression was once again confined to the epithelium. In contrast to the strong expression of FTT ζ in primary corneal epithelial cells, a low expression of this isoform was evident in primary conjunctival epithelial cells (Figure 3). This apparent “differential” expression, based on quantified western blot analyses of cultured cell lysates, suggests that the interaction of FTT isoforms with epithelial proliferation/differentiation machinery may exhibit some epithelial tissue preference. We therefore suggest that while FTT ζ may be preferentially expressed by the corneal epithelium, FTT γ expression is characteristic of the conjunctival epithelium. In contrast to the qualitative immunofluorescence data that represent in vivo status, quantified western blot analyses of cell lysates may reflect the influence of the in vitro environment. Lack of access to SV40 and E6/E7 immortalized conjunctival epithelial cell lines prevented us from comparing the FTT expression profile in primary conjunctival epithelial cells (e.g., low expression of FTT ζ isoform) with that in hyperproliferative cell lines. It was more difficult to precisely assign distribution of isoform expression within the conjunctival epithelium because we had no information about tissue orientation of the eye bank tissue.

It has also been shown that FTT σ is secreted in the culture medium during propagation of an SV40 immortalized corneal cell line [18]. We show for the first time that primary corneal and conjunctival epithelial cells and E6/E7 and SV40 immortalized corneal epithelial cells secrete FTT σ as well FTT γ and ζ isoforms when cultured in a serum free defined medium. Although the intracellular functions of FTT proteins are beginning to be better understood, the role of secreted isoforms is mostly obscure. It has been shown that recombinant FTT σ upregulates secretion of matrix metalloproteinases (MMPs) by normal human dermal fibroblasts [57] and that the exosomal export is a minor component of the overall secretion mechanisms [57]. The extracellular function of FTT γ and ζ isoforms, the major export mechanisms of FTT σ from the secreting cells, and the mechanism of import into and the nature of interaction of the secreted proteins with the target cells are not known [15]. The functions of MMPs in tissue homeostasis are regulated by the appropriate tissue inhibitors of matrix metalloproteinases (TIMPs). This equilibrium is altered after injury and during disease processes where matrix degradation and reorganization (remodeling) are likely to take place [58]. Since injuries to the ocular surface increase signaling events that control the responses and “cross-talk” between the epithelium and the stroma, it is likely that the excreted FTT isoforms play a more important role during the wound healing/tissue repair process.

The interaction of the epithelia with the tear film compartment is also important in the homeostasis of the corneal and conjunctival epithelia. The cells of all the anterior segment tissues contribute to the tear film composition while the tear fluid components combine to protect the epithelia from microbial and chemical insults and help maintain epithelial barrier structure and functions. Therefore, we examined the human tear fluid for the presence of FTT isoforms, which we found to be secreted into the condition medium in vitro. Although FTT σ could not be detected, FTT γ and ζ isoforms were present. Since the primary conjunctival epithelial cells express “preferentially” FTT γ while FTT ζ is preferentially expressed by the primary corneal epithelial cells, we speculate that γ and ζ isoforms are secreted by the conjunctival and corneal epithelia, respectively. FTT σ is expressed abundantly in the superficial layers of the corneal and conjunctival epithelium. Since it is absent from the tear fluid, it is unlikely that the presence of FTT γ and ζ is due to the lysis of corneal and conjunctival epithelial cells.

We report here the expression and distribution of all the FTT isoforms in the human cornea and conjunctiva. Of particular interest is the expression specificity of FTT σ in the corneal and the conjunctival epithelia and its potential association with the epithelial cell differentiation. We have also shown the presence of FTT proteins in the corneal and conjunctival primary cells and corneal cell lines and report the secretion of three isoforms (σ, γ, and ζ) by the primary epithelial cells and cell lines. Interestingly, of the three secreted isoforms, only FTT γ and ζ were found to be present in tear fluid from healthy human donors. We have also shown low expression of FTT θ in the primary corneal epithelial cells when compared with the cell lines and note the preferential expression of FTT γ and FTT ζ in the primary corneal and conjunctival cells, respectively. In studying the functional implications of the above observation, we are particularly interested in the functional roles of the secreted isoforms found in the tear fluid. Taken together, our results show the presence and support the importance of the FTT family of proteins in the tissues of the human ocular surface and indicate the need for a better understanding of their function in the ocular surface homeostasis and repair.

## References

[1] Kjarland E, Keen TJ, Kleppe R (2006). Does isoform diversity explain functional differences in the 14–3-3 protein family?. Curr Pharm Biotechnol.

[2] Muslin AJ, Xing H (2000). 14–3-3 proteins: regulation of subcellular localization by molecular interference.. Cell Signal.

[3] Dougherty MK, Morrison DK (2004). Unlocking the code of 14–3-3.. J Cell Sci.

[4] Mhawech P (2005). 14–3-3 proteins–an update.. Cell Res.

[5] Aitken A (2006). 14–3-3 proteins: a historic overview.. Semin Cancer Biol.

[6] Henriksson ML, Francis MS, Peden A, Aili M, Stefansson K, Palmer R, Aitken A, Hallberg B (2002). A nonphosphorylated 14–3-3 binding motif on exoenzyme S that is functional in vivo.. Eur J Biochem.

[7] Zhai J, Lin H, Shamim M, Schlaepfer WW, Canete-Soler R (2001). Identification of a novel interaction of 14–3-3 with p190 Rho GEF.. J Biol Chem.

[8] Petosa C, Masters SC, Bankston LA, Pohl J, Wang B, Fu H, Liddington RC (1998). 14–3-3 zeta binds a phosphorylated Raf peptide and an unphosphorylated peptide via its conserved amphipathic groove.. J Biol Chem.

[9] Masters SC, Pederson KJ, Zhang L, Barbieri JT, Fu H (1999). Interaction of 14–3-3 with a nonphosphorylated protein ligand, exoenzyme S of Pseudomonas aeruginosa.. Biochemistry.

[10] Zhang L, Wang H, Masters SC, Wang B, Barbieri JT, Fu H (1999). Residues of 14–3-3 zeta required for activation of exoenzyme S of Pseudomonas aeruginosa.. Biochemistry.

[11] Hermeking H, Benzinger A (2006). 14–3-3 proteins in cell cycle regulation.. Semin Cancer Biol.

[12] van Heusden GP (2005). 14–3-3 proteins: regulators of numerous eukaryotic proteins.. IUBMB Life.

[13] Ghahary A, Karimi-Busheri F, Marcoux Y, Li Y, Tredget EE, Taghi Kilani R, Li L, Zheng J, Karami A, Keller BO, Weinfeld M (2004). Keratinocyte-releasable stratifin functions as a potent collagenase-stimulating factor in fibroblasts.. J Invest Dermatol.

[14] Lam E, Kilani RT, Li Y, Tredget EE, Ghahary A (2005). Stratifin-induced matrix metalloproteinase-1 in fibroblast is mediated by c-fos and p38 mitogen-activated protein kinase activation.. J Invest Dermatol.

[15] Ghahary A, Marcoux Y, Karimi-Busheri F, Li Y, Tredget EE, Kilani RT, Lam E, Weinfeld M (2005). Differentiated keratinocyte-releasable stratifin (14–3-3 sigma) stimulates MMP-1 expression in dermal fibroblasts.. J Invest Dermatol.

[16] Ghaffari A, Li Y, Karami A, Ghaffari M, Tredget EE, Ghahary A (2006). Fibroblast extracellular matrix gene expression in response to keratinocyte-releasable stratifin.. J Cell Biochem.

[17] Kilani RT, Guilbert L, Lin X, Ghahary A (2007). Keratinocyte conditioned medium abrogates the modulatory effects of IGF-1 and TGF-beta1 on collagenase expression in dermal fibroblasts.. Wound Repair Regen.

[18] Zanello SB, Nayak R, Zanello LP, Farthing-Nayak P (2006). Identification and distribution of 14–3-3 sigma (stratifin) in the human cornea.. Curr Eye Res.

[19] Vercoutter-Edouart AS, Lemoine J, Le Bourhis X, Louis H, Boilly B, Nurcombe V, Revillion F, Peyrat JP, Hondermarck H (2001). Proteomic analysis reveals that 14–3-3 sigma is down-regulated in human breast cancer cells.. Cancer Res.

[20] Moreira JM, Gromov P, Celis JE (2004). Expression of the tumor suppressor protein 14–3-3 sigma is down-regulated in invasive transitional cell carcinomas of the urinary bladder undergoing epithelial-to-mesenchymal transition.. Mol Cell Proteomics.

[21] Cheng L, Pan CX, Zhang JT, Zhang S, Kinch MS, Li L, Baldridge LA, Wade C, Hu Z, Koch MO, Ulbright TM, Eble JN (2004). Loss of 14–3-3 sigma in prostate cancer and its precursors.. Clin Cancer Res.

[22] Uchida D, Begum NM, Almofti A, Kawamata H, Yoshida H, Sato M (2004). Frequent downregulation of 14–3-3 sigma protein and hypermethylation of 14–3-3 sigma gene in salivary gland adenoid cystic carcinoma.. Br J Cancer.

[23] Tzivion G, Gupta VS, Kaplun L, Balan V (2006). 14–3-3 proteins as potential oncogenes.. Semin Cancer Biol.

[24] Toyooka K, Muratake T, Watanabe H, Hayashi S, Ichikawa T, Usui H, Washiyama K, Kumanishi T, Takahashi Y (2002). Isolation and structure of the mouse 14–3-3 eta chain gene and the distribution of 14–3-3 eta mRNA in the mouse brain.. Brain Res Mol Brain Res.

[25] Li Q, Lu Q, Estepa G, Verma IM (2005). Identification of 14–3-3sigma mutation causing cutaneous abnormality in repeated-epilation mutant mouse.. Proc Natl Acad Sci USA.

[26] Herron BJ, Liddell RA, Parker A, Grant S, Kinne J, Fisher JK, Siracusa LD (2005). A mutation in stratifin is responsible for the repeated epilation (Er) phenotype in mice.. Nat Genet.

[27] Moore JE, McMullen CB, Mahon G, Adamis AP (2002). The corneal epithelial stem cell.. DNA Cell Biol.

[28] Pellegrini G, Dellambra E, Golisano O, Martinelli E, Fantozzi I, Bondanza S, Ponzin D, McKeon F, De Luca M (2001). p63 identifies keratinocyte stem cells.. Proc Natl Acad Sci USA.

[29] Dimitrijevich SD, Reese TJ, Gracy RW, Oakford LX, Howe WE. A Transmission Electron Microscopic Study of Methods for Harvesting Epithelial Cells from Human Corneal Tissue. In, Bailey, GW and Hall EL eds., Proceedings: Electron Microscopy Soc. of America, 49th Ann. Meeting 1991, p174, San Francisco Press, San Francisco.

[30] Dimitrijevich SD, Reese TJ, Gracy RW, Wordinger R (1993). FGF (a,b) in the human cornea and the epithelial tissue equivalent.. Invest Ophthalmol Vis Sci.

[31] Dimitrijevich SD, Gracy R.W. Non-Contracting Tissue Equivalents (Skin, Cornea and Conjunctiva), (US Pat. 6,471,958, October, 2002).

[32] Offord EA, Sharif NA, Mace K, Tromvoukis Y, Spillare EA, Avanti O, Howe WE, Pfeifer AM (1999). Immortalized human corneal epithelial cells for ocular toxicity and inflammation studies.. Invest Ophthalmol Vis Sci.

[33] Wilson SE, Weng J, Blair S, He YG, Lloyd S (1995). Expression of E6/E7 or SV40 large T antigen-coding oncogenes in human corneal endothelial cells indicates regulated high-proliferative capacity.. Invest Ophthalmol Vis Sci.

[34] Dimitrijevich SD, Reese TJ, Yorio T, Gracy RW (1994). In vitro model of the human corneal endothelium.. Invest Ophthalmol Vis Sci.

[35] Argueso P, Balaram M, Spurr-Michaud S, Keutmann HT, Dana MR, Gipson IK (2002). Decreased levels of the goblet cell mucin MUC5AC in tears of patients with Sjogren syndrome.. Invest Ophthalmol Vis Sci.

[36] Spurr-Michaud S, Argueso P, Gipson I (2007). Assay of mucins in human tear fluid.. Exp Eye Res.

[37] Dimitrijevich SD, Reese TJ, Yorio T, Gracy RW (1995). An in vitro model of the human cornea.. Invest Ophthalmol Vis Sci.

[38] Ward SL, Walker TL, Dimitrijevich SD (1997). Evaluation of Chemically-Induced Toxicity Using an In Vitro Model of Human Corneal Epithelium.. Toxicol In Vitro.

[39] Gamache DA, Dimitrijevich SD, Weimer LK, Lang LS, Spellman JM, Graff G, Yanni JM (1997). Secretion of proinflammatory cytokines by human conjunctival epithelial cells.. Ocul Immunol Inflamm.

[40] Matic M, Petrov IN, Chen S, Wang C, Dimitrijevich SD, Wolosin JM (1997). Stem cells of the corneal epithelium lack connexins and metabolite transfer capacity.. Differentiation.

[41] Dellambra E, Golisano O, Bondanza S, Siviero E, Lacal P, Molinari M, D'Atri S, De Luca M (2000). Downregulation of 14–3-3 sigma prevents clonal evolution and leads to immortalization of primary human keratinocytes.. J Cell Biol.

[42] Kirschner M, Montazem A, Hilaire HS, Radu A (2006). Long-term culture of human gingival keratinocyte progenitor cells by down-regulation of 14–3-3 sigma.. Stem Cells Dev.

[43] Shen J, Pavone A, Mikulec C, Hensley SC, Traner A, Chang TK, Person MD, Fischer SM (2007). Protein expression profiles in the epidermis of cyclooxygenase-2 transgenic mice by 2-dimensional gel electrophoresis and mass spectrometry.. J Proteome Res.

[44] Akahira J, Sugihashi Y, Suzuki T, Ito K, Niikura H, Moriya T, Nitta M, Okamura H, Inoue S, Sasano H, Okamura K, Yaegashi N (2004). Decreased expression of 14–3-3 sigma is associated with advanced disease in human epithelial ovarian cancer: its correlation with aberrant DNA methylation.. Clin Cancer Res.

[45] Guweidhi A, Kleeff J, Giese N, El Fitori J, Ketterer K, Giese T, Buchler MW, Korc M, Friess H (2004). Enhanced expression of 14–3-3sigma in pancreatic cancer and its role in cell cycle regulation and apoptosis.. Carcinogenesis.

[46] Hermeking H (2003). The 14–3-3 cancer connection.. Nat Rev Cancer.

[47] Ito Y, Miyoshi E, Uda E, Yoshida H, Uruno T, Takamura Y, Miya A, Kobayashi K, Matsuzuka F, Matsuura N, Kakudo K, Kuma K, Miyauchi A (2003). 14–3-3 sigma possibly plays a constitutive role in papillary carcinoma, but not in follicular tumor of the thyroid.. Cancer Lett.

[48] Seimiya H, Sawada H, Muramatsu Y, Shimizu M, Ohko K, Yamane K, Tsuruo T (2000). Involvement of 14–3-3 proteins in nuclear localization of telomerase.. EMBO J.

[49] Bonin LR, Madden K, Shera K, Ihle J, Matthews C, Aziz S, Perez-Reyes N, McDougall JK, Conroy SC (1999). Generation and characterization of human smooth muscle cell lines derived from atherosclerotic plaque.. Arterioscler Thromb Vasc Biol.

[50] Yang Y, Spector A, Ma W, Wang RR, Larsen K, Kleiman NJ (1998). The effect of catalase amplification on immortal lens epithelial cell lines.. Exp Eye Res.

[51] Dickson MA, Hahn WC, Ino Y, Ronfard V, Wu JY, Weinberg RA, Louis DN, Li FP, Rheinwald JG (2000). Human keratinocytes that express hTERT and also bypass a p16(INK4a)-enforced mechanism that limits life span become immortal yet retain normal growth and differentiation characteristics.. Mol Cell Biol.

[52] Dimitrijevich SD, Boswell G, Ramirez RD, Shay JW (2001). Telomerase Transfected Human Corneal Epithelial Cells as Components of Tissue Equivalents.. Invest Ophthalmol Vis Sci.

[53] Jester JV, Huang J, Fisher S, Spiekerman J, Chang JH, Wright WE, Shay JW (2003). Myofibroblast differentiation of normal human keratocytes and hTERT, extended-life human corneal fibroblasts.. Invest Ophthalmol Vis Sci.

[54] Egan CA, Savre-Train I, Shay JW, Wilson SE, Bourne WM (1998). Analysis of telomere lengths in human corneal endothelial cells from donors of different ages.. Invest Ophthalmol Vis Sci.

[55] Pereira-Faca SR, Kuick R, Puravs E, Zhang Q, Krasnoselsky AL, Phanstiel D, Qiu J, Misek DE, Hinderer R, Tammemagi M, Landi MT, Caporaso N, Pfeiffer R, Edelstein C, Goodman G, Barnett M, Thornquist M, Brenner D, Hanash SM (2007). Identification of 14–3-3 theta as an antigen that induces a humoral response in lung cancer.. Cancer Res.

[56] Martínez-Galán J, Torres B, Del Moral R, Muñoz-Gámez JA, Martín-Oliva D, Villalobos M, Núñez MI, Luna Jde D, Oliver FJ, Ruiz de Almodóvar JM (2008). Quantitative detection of methylated ESR1 and 14-3-3-sigma gene promoters in serum as candidate biomarkers for diagnosis of breast cancer and evaluation of treatment efficacy.. Cancer Biol Ther.

[57] Chavez-Munoz C, Morse J, Kilani R, Ghahary A (2008). Primary human keratinocytes externalize stratifin protein via exosomes.. J Cell Biochem.

[58] Nagase H, Visse R, Murphy G (2006). Structure and function of matrix metalloproteinases and TIMPs.. Cardiovasc Res.

